# The Effects of Dipeptidyl Peptidase 4 Inhibitors on Renal Function in Patients with Type 2 Diabetes Mellitus

**DOI:** 10.3390/jcm11092653

**Published:** 2022-05-09

**Authors:** Wan-Chia Hsu, Chun-Sheng Lin, Jung-Fu Chen, Chih-Min Chang

**Affiliations:** Division of Endocrinology and Metabolism, Department of Internal Medicine, Kaohsiung Chang Gung Memorial Hospital, Kaohsiung 833, Taiwan; kaimu9518@cgmh.org.tw (W.-C.H.); yyy555385@gmail.com (C.-S.L.); 0722cjf@cgmh.org.tw (J.-F.C.)

**Keywords:** diabetic kidney disease, DPP-4 inhibitor, eGFR, renal function, type 2 diabetes mellitus

## Abstract

Past studies have confirmed that glucagon-like peptide 1 (GLP-1) receptor agonists can improve renal outcomes in patients with type 2 diabetes mellitus (DM). This study aimed to evaluate whether dipeptidyl peptidase 4 (DPP-4) inhibitors, which elevate GLP-1 levels, also have similar effects on renal function. In this retrospective study, diabetic patients treated with anti-hyperglycemic agents between 2008 and 2011 were selected. We compared the time to first occurrence of estimated glomerular filtration rate (eGFR) decline ≥30% from the baseline between patients treated with DPP-4 inhibitors and those treated with other anti-hyperglycemic drugs. A total of 2202 patients were enrolled. The incidence of eGFR decline ≥30% from the baseline was 10.08% in the DPP-4 inhibitor group and 16.17% in the non-DPP-4 inhibitor group (*p* < 0.001). The mean time to event was significantly longer in patients receiving DPP-4 inhibitors (2.84 ± 1.60 vs. 1.96 ± 1.30 years, *p* < 0.001). Patients who were younger than 65 years old, had better baseline eGFR, did not have preexisting hyperlipidemia, or who were untreated with concomitant statin showed greater reductions in the risk of renal function decline (all *p* for interaction < 0.05). Conclusively, DPP-4 inhibitors used alone or in combination with other glucose-lowering agents were correlated with lower risks of eGFR decline in patients with type 2 DM.

## 1. Introduction

In 2021, approximately 537 million adults aged 20–79 years were estimated to be living with diabetes mellitus (DM) worldwide [1]. The global prevalence of DM continues to rise; this number is predicted to reach 643 million or 11.3% of the adult population by 2030 and 783 million or 12.2% by 2045 [1]. In 2021, DM and its complications were responsible for approximately 6.7 million deaths [1]. The total health expenditure on DM is approximately USD 966 billion, and is expected to grow, posing considerable social and economic burden [1].

Diabetic kidney disease (DKD) is a long-term vascular complication associated with DM, occurring in 20–40% of diabetic patients [2,3]. DKD is the leading cause of end-stage renal disease (ESRD) in the United States, and increases the risk of cardiovascular events, as well as healthcare costs [3,4]. Therefore, it is important to prevent the new-onset of DKD or slow its progression.

Over the past few years, several studies have suggested that sodium-glucose cotransporter 2 (SGLT2) inhibitors and glucagon-like peptide 1 receptor agonists (GLP-1 RAs) have beneficial effects on renal outcomes in patients with type 2 DM [5,6,7,8,9,10,11]. Dipeptidyl peptidase 4 (DPP-4) inhibitors are also incretin-based therapies for type 2 DM and have the same glucose-lowering mechanism as GLP-1 RAs. In this study, we aimed to assess whether DPP-4 inhibitors exhibit similar effects on renal function as GLP-1 RAs.

## 2. Materials and Methods

### 2.1. Study Design and Study Population

This was a retrospective study conducted with data obtained from the Chang Gung Research Database (CGRD), an electronic medical record of patients from Chang Gung Memorial Hospital (CGMH). CGMH is a medical center that provides a range of medical services through 10 medical institutes located across Taiwan. The information from the CGRD database detailed the sex, birth, diagnosis, medications, laboratory test results, and imaging data for all inpatients, outpatients, and patients visiting the emergency department since 2001. The database is updated monthly. This study was approved by the Chang Gung Medical Foundation Institutional Review Board (permit number: 201801059B0).

We enrolled patients who were aged ≥18 years, diagnosed with type 2 DM, and treated with anti-hyperglycemic agents between 1 January 2008 and 31 December 2011. The diagnosis of the underlying comorbidity was defined according to the International Classification of Diseases (ICD)-9 code. Patients with an established history of, renal failure (in accordance with ICD-9 code 585), or kidney transplantation were excluded from this study. Only serum creatinine levels were documented directly in the CGRD; therefore, the estimated glomerular filtration rate (eGFR) was calculated using the 4-Variable Modification of Diet in Renal Disease (MDRD) equation. Patients in whom the baseline eGFR value, averaged from the last three records before the index date, was below 15 mL/min/1.73 m^2^ were excluded from the study. Baseline glycohemoglobin (HbA1c) was taken as the mean of the last two or three records before the index date. Patients for whom the eGFR or HbA1c data prior to the index date or eGFR data during the follow-up period were incomplete were excluded from the study. Additionally, patients receiving treatment with insulin, insulin analogues, SGLT2 inhibitors, or GLP-1 RAs before the index date and during the follow-up period were excluded.

The patients were divided into two groups based on the initial drug prescriptions: those treated with DPP-4 inhibitors and those treated with other anti-hyperglycemic agents. DPP-4 inhibitors, including sitagliptin, vildagliptin, saxagliptin, linagliptin, alogliptin, and compounds containing any of these contents, may be prescribed four times in a year. Therefore, we defined DPP-4 inhibitor users as those who received prescriptions for DPP-4 inhibitors at least three times in every follow-up year, alone or in combination with other classes of anti-hyperglycemic agents, on the basis of medication possession rates of ≥60%. The first date of prescription of DPP-4 inhibitors or other anti-hyperglycemic medications was defined as the index date. The total observation period was 5 years.

To reduce potential selection bias, the two groups were matched using a propensity score, ensuring a 1:1 ratio of age, sex, baseline eGFR, baseline HbA1c, underlying comorbidities (including coronary artery disease, heart failure, cerebrovascular disease, hyperlipidemia, hypertension, gout, and obesity), and prior medications (including angiotensin converting enzyme inhibitors (ACEIs) or angiotensin receptor blockers (ARBs), direct renin inhibitors, statins, antihypertensive agents, and anti-gout drugs). Prior medications were defined as ≥28 days of use within 365 days before index date.

### 2.2. Outcomes

The renal outcome was defined as the time to the first occurrence of eGFR decline ≥30% from the baseline. We evaluated eGFR values annually, and averaged the values from the last 1 to 3 records before the date of evaluation for comparison. Additionally, renal outcomes were evaluated by stratification of the baseline CKD stages. The CKD stages were classified as 1 to 5, representing eGFR ≥ 90 mL/min/1.73 m^2^, eGFR ≥ 60 to <90 mL/min/1.73 m^2^, eGFR ≥ 30 to <60 mL/min/1.73 m^2^, eGFR ≥ 15 to <30 mL/min/1.73 m^2^, and eGFR < 15 mL/min/1.73 m^2^, respectively, as per the Kidney Disease: Improving Global Outcomes (KDIGO) guidelines [12].

### 2.3. Statistical Analyses

For continuous variables, the means and standard deviations (SDs) are reported. For categorical variables, data are presented as numbers and percentages. The differences between the two groups were compared using the independent sample t-test for continuous variables and the Chi-square test for categorical variables. 

All patients were followed up until the outcome of interest, loss of follow-up, or the latest date of this study, depending on which occurred first. Cumulative incidences were estimated using the Kaplan–Meier method and log-rank test for *p*-values. The Cox proportional hazard regression model was used to calculate hazard ratios (HRs) and the 95% confidence interval (CI) was used to evaluate the effect of the variables or intervention on the outcome of the study. The effect of the variables or intervention on the outcome was tested in subgroups of age, gender, baseline eGFR, baseline HbA1c, underlying comorbidities, and concomitant use of medication. Concomitant medication use was defined as that prescribed for ≥28 days after the index date, until the first date of occurrence of events. Statistical significance was set at *p* < 0.05. All statistical data were analyzed using the SAS software, version 9.4 (SAS Institute Inc., Cary, NC, USA).

## 3. Results

### 3.1. Study Patients and Baseline Characteristics

**Figure 1:**
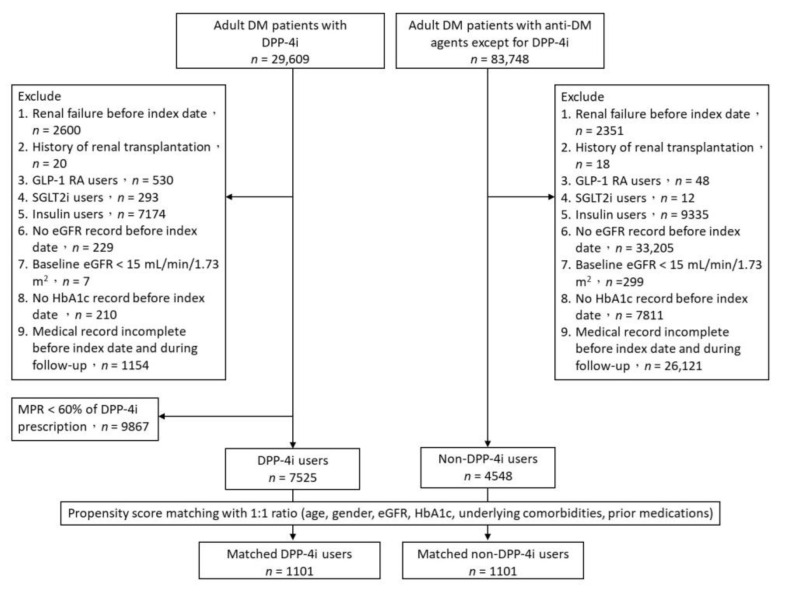
Flow chart of patient selection. DM: diabetes mellitus; DPP-4i: dipeptidyl peptidase 4 inhibitor; GLP-1 RA: glucagon-like peptide 1 receptor agonist; SGLT2i: sodium-glucose cotransporter 2 inhibitors; eGFR: estimated glomerular filtration rate; HbA1c: glycohemoglobin; MPR: medication possession rate.

Records from 1 January 2008 to 31 December 2011 for a total of 113,357 patients aged between 18 and 89 years with type 2 DM were identified, of which 12,073 satisfied the inclusion and exclusion criteria. Of these, 7525 patients were treated with DPP-4 inhibitors and 4548 were treated with other anti-hyperglycemic agents. Propensity score matching with a 1:1 ratio was applied, and records for 1101 DPP-4 inhibitor users and 1101 matched non-DPP-4 inhibitor users were selected for analysis (Figure 1). 

The demographic and baseline clinical characteristics of the two groups of patients were not significantly different (Table 1). The mean ages of patients in the DPP-4 inhibitor group and non-DPP inhibitor group were 63.18 ± 11.18 years and 63.43 ± 12.62 years, respectively. Of the DPP-4 inhibitor and non-DPP-4 inhibitor users, 54.13 and 53.95% were male, respectively. The mean eGFR of patients in the DPP-4 inhibitor group was slightly higher than that of patients in the non-DPP-4 inhibitor group (77.84 ± 27.56 mL/min/1.73 m^2^ vs. 77.60 ± 27.19 mL/min/1.73 m^2^). The baseline HbA1c level was mildly lower in the DPP-4 inhibitor group than in the other group (7.59 ± 1.24% vs. 7.63 ± 1.47%). The most common drugs recorded in the prior medications were antihypertensive drugs, which were prescribed to 67.94% of the patients in the DPP-4 inhibitor group and to 68.66% in the non-DPP-4 inhibitor group. ACEIs or ARBs were also taken by approximately half of the patients in both groups (51.77 vs. 49.14%). Most patients in both groups had hyperlipidemia (58.13 vs. 57.77%) and hypertension (73.48 vs. 74.93%).

### 3.2. Outcomes

**Figure 2:**
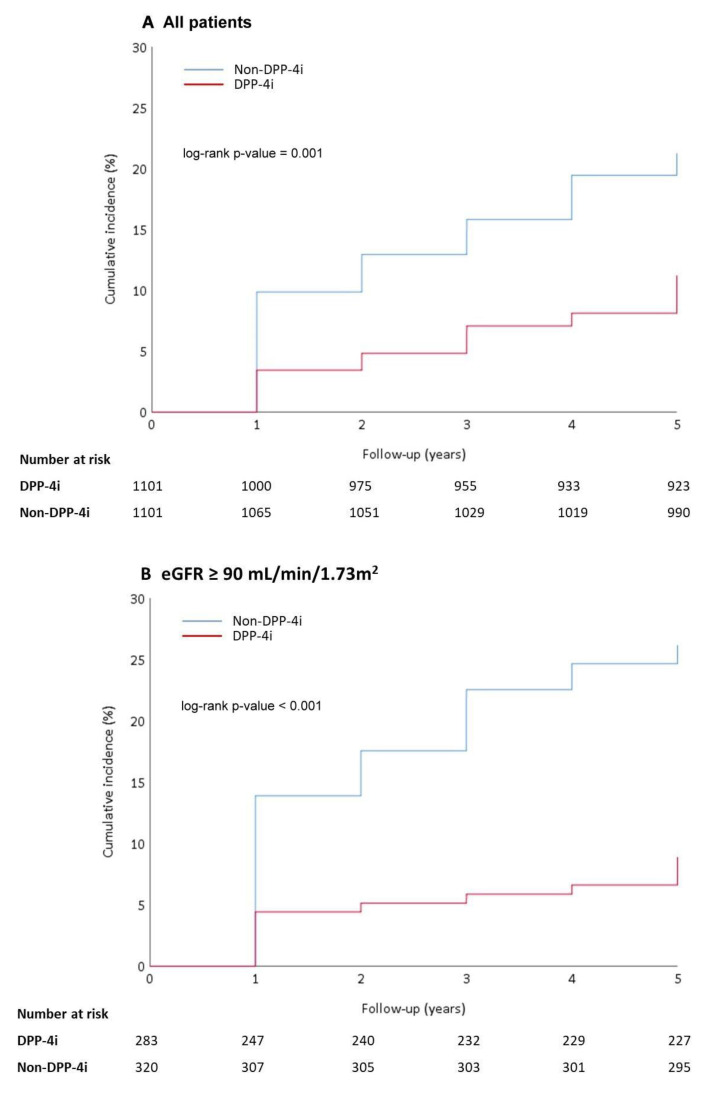

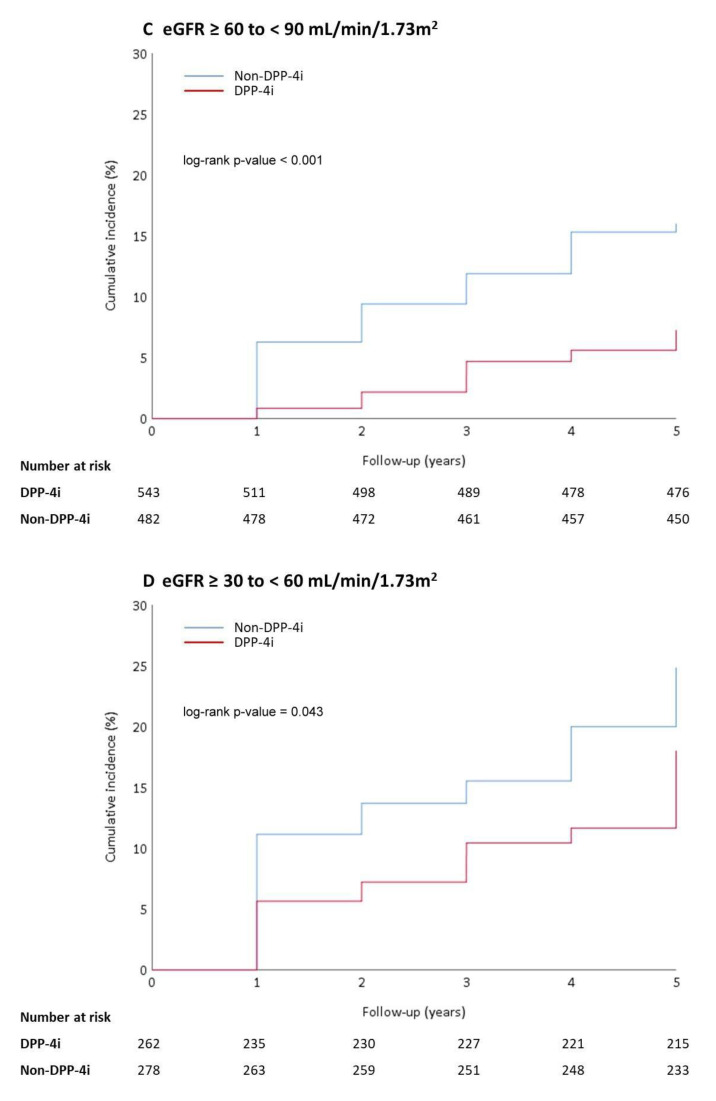

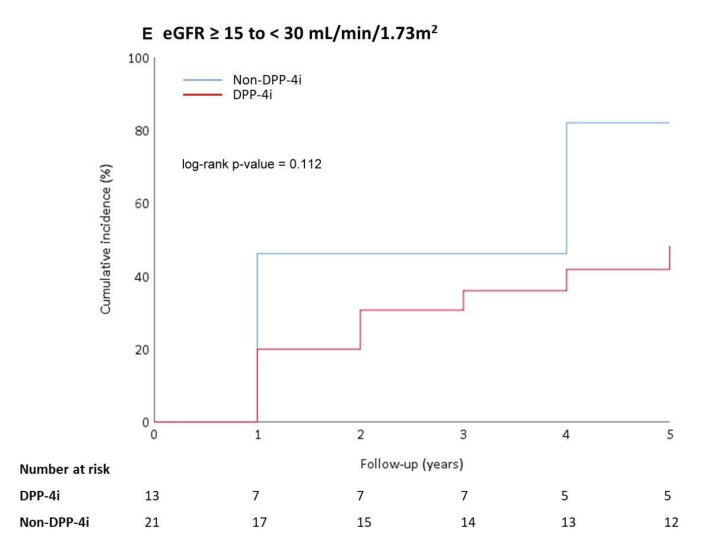
Cumulative incidence rates of eGFR decline ≥ 30% from the baseline between the DPP-4 inhibitor group and non-DPP-4 inhibitor group shown by Kaplan–Meier curves: (**A**) all patients; (**B**) patients with baseline eGFR ≥ 90 mL/min/1.73 m^2^; (**C**) patients with baseline eGFR ≥ 60 to <90 mL/min/1.73 m^2^; (**D**) patients with baseline eGFR ≥ 30 to < 60 mL/min/1.73 m^2^; (**E**) patients with baseline eGFR ≥ 15 to < 30 mL/min/1.73 m^2^. DPP-4i: dipeptidyl peptidase 4 inhibitor; eGFR: estimated glomerular filtration rate.

During the 5-year follow-up period, the incidence of eGFR decline by ≥30% from the baseline was 10.08% in the DPP-4 inhibitor group, which was significantly lower (*p* < 0.001) than that in the non-DPP-4 inhibitor group (16.17%) (Table 2). The mean time to event was observed to be significantly longer in patients treated with DPP-4 inhibitors than in those treated with other anti-hyperglycemic agents (2.84 ± 1.60 years vs. 1.96 ± 1.30 years, *p* < 0.001) (Table 2). The cumulative incidence rates of eGFR decline ≥ 30% were also significantly different between the two groups during the 5-year follow-up, with a higher incidence in the non-DPP-4 inhibitor group (log-rank test, *p* = 0.001) (Figure 2A). The event rate of eGFR decline ≥30%, categorized by baseline CKD stage, is demonstrated in Figure 2B–E. Regardless of the baseline CKD stage, patients treated with DPP-4 inhibitors had a lower incidence of eGFR decline ≥30% during the 5-year period. However, this difference between the two groups decreased as the baseline CKD stage increased, with no significant difference between the groups being recorded at baseline CKD stage 4 (log-rank test, *p* = 0.112) (Figure 2E).

**Figure 3:**
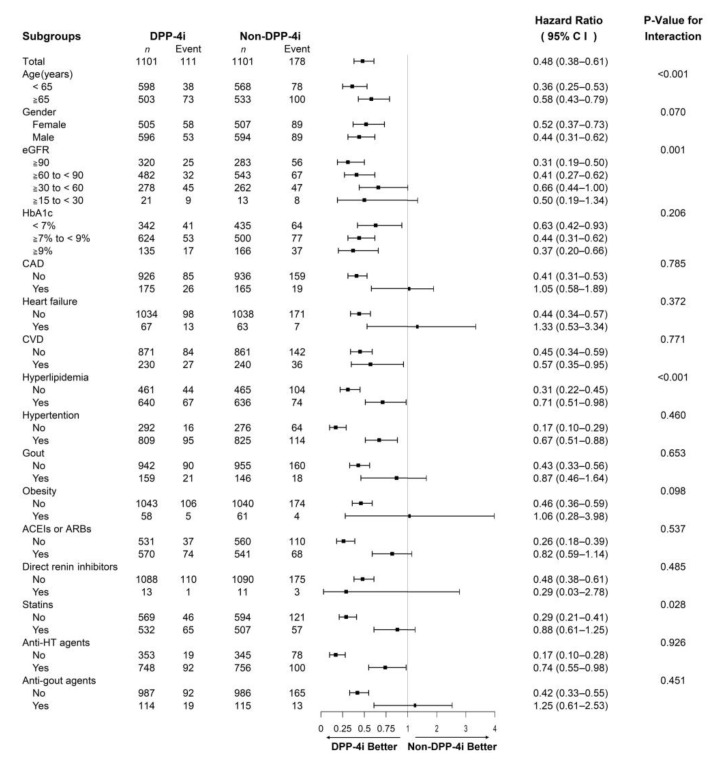
Subgroup analyses of renal outcomes. The hazard ratio and 95% confidence interval (CI) were estimated with the Cox proportional hazard regression model. DPP-4i: dipeptidyl peptidase 4 inhibitor; eGFR: estimated glomerular filtration rate; HbA1c: glycohemoglobin; CAD: coronary artery disease; CVD: cerebrovascular disease; ACEIs: angiotensin converting enzyme inhibitors; ARBs: angiotensin receptor blockers; anti-HT: antihypertensive.

The risk of eGFR decline ≥ 30% was observed to be over 50% lower in diabetic patients treated with DPP-4 inhibitors than in those treated with other drugs (HR = 0.48, 95% CI: 0.38–0.61). The subgroup analyses for renal function decline are shown in Figure 3. 

The risks were significantly reduced among patients treated with DPP-4 inhibitors, regardless of their age (<65 or ≥65 years); sex; HbA1c level (<7%, ≥7 to <9%, or ≥9%); concomitant use of antihypertensive drugs; or underlying comorbidities of cerebrovascular disease, hyperlipidemia, and hypertension. And the younger patients below the age of 56 years presented a lower risk than the older patients (<65 years, HR = 0.36, 95% CI: 0.25–0.53; ≥65 years, HR = 0.58, 95% CI: 0.43–0.79; *p* for interaction < 0.001). 

DPP-4 inhibitor users were at a lower risk of eGFR decline ≥ 30%, irrespective of the baseline CKD stage, but this difference was significant only when the baseline eGFR was ≥60 mL/min/1.73 m^2^ (eGFR ≥ 90 mL/min/1.73 m^2^, HR = 0.31, 95% CI: 0.19–0.50; eGFR ≥ 60 to < 90 mL/min/1.73 m^2^, HR = 0.41, 95% CI: 0.27–0.62). In addition, there was a decreasing trend of the risks of renal function decline in patients with better baseline eGFR (eGFR ≥ 90 mL/min/1.73 m^2^, HR = 0.31, 95% CI: 0.19–0.50; eGFR ≥ 60 to < 0 mL/min/1.73 m^2^, HR = 0.41, 95% CI: 0.27–0.62; eGFR ≥ 30 to < 60 mL/min/1.73 m^2^, HR = 0.66, CI: 0.44–1.00; eGFR ≥ 15 to < 30 mL/min/1.73 m^2^, HR = 0.50, CI: 0.19–1.34; *p* for interaction = 0.001).

Patients without concomitant medication or underlying comorbidities, upon treatment with DPP-4 inhibitors, showed a decreased susceptibility to decline in renal function. However, the risks of eGFR decline were significantly lower among patients without hyperlipidemia (*p* for interaction < 0.001) or untreated with statins (*p* for interaction = 0.028).

## 4. Discussion

This retrospective study was conducted with a longer follow-up period and more patients involved. Our study demonstrated that adult patients diagnosed with type 2 DM and treated with DPP-4 inhibitors, alone or in combination with other anti-hyperglycemic agents, presented lower risks of eGFR decline than those treated with other anti-hyperglycemic agents.

DM is a chronic disease associated with macrovascular (coronary artery disease, stroke, and peripheral artery disease) and microvascular (retinopathy, nephropathy, and neuropathy) complications. Cardiovascular disease is a major cause of death in individuals with DM, accounting for approximately 65% of the deaths in diabetic patients [13,14]. DM-related microvascular complications may result in blindness, amputation, and ESRD, requiring life-long dialysis [3,14]. The healthcare costs of treatment of these complications are high, accounting for more than 50% of the direct health costs for DM [1]. Therefore, strategies to mitigate the long-term complications of DM are the cornerstone of treatments for patients with DM. Previous large prospective studies have demonstrated that optimizing glycemic control can decrease the risks of macro- and microvascular complications in patients with type 2 DM [3,15,16,17]. In recent years, several cardiovascular outcome trials have demonstrated that SGLT2 inhibitors and GLP-1 RAs can substantially reduce cardiovascular events in patients with type 2 DM, independent of their glucose-lowering effect [5,6,7,9,11,18]. The beneficial effects of SGLT2 inhibitors and GLP-1 RAs on renal outcomes have also been reported [5,6,7,8,9,10,11]. 

GLP-1 is an incretin peptide hormone. It is secreted by the L-cells of the small intestine in response to food intake and stimulates insulin secretion in a glucose-dependent manner, while suppressing glucagon release from pancreatic α-cells. In pancreatic β-cells, GLP-1 enhances glucose sensitivity and β-cell proliferation and decreases β-cell apoptosis. Furthermore, GLP-1 slows down gastric emptying and small intestinal peristalsis, while increasing central satiety and reducing appetite. These actions contribute to the amelioration of glycemic control and reductions in body weight [19,20]. DPP-4 inhibitors are also an incretin-based therapy for DM. These drugs inhibit the enzyme DPP-4, which is responsible for the degradation of GLP-1. Therefore, these drugs help maintain serum GLP-1 concentration, and subsequently lower blood glucose levels and promote relevant physiologic effects [21].

Notably, GLP-1 also directly affects kidney function and is not mediated through glycemia. GLP-1 has been reported to increase natriuresis and diuresis [22]. The kidney transporter thought to mediate these effects is the sodium–hydrogen exchanger 3 (NHE3), which is located at the brush border of the renal proximal tubular cells and bound to a complex that also contains DPP-4. This action may partially explain the blood-pressure-lowering effect of GLP-1 RAs, which also influences renal function [20]. Moreover, there is increasing evidence suggesting that inflammatory cells, cytokines, and profibrotic growth factors play a role in the pathogenesis of DKD by increasing vascular inflammation and fibrosis. Preclinical studies have found that GLP-1 may be able to protect the kidneys from hyperglycemia-induced oxidative stress by activating the cyclic adenosine monophosphate–protein kinase A (cAMP–PKA) pathway, leading to an increase in cAMP and a subsequent reduction in the levels of nicotinamide adenine dinucleotide phosphate (NAD(P)H) oxidase [20,23].

DPP-4 inhibitors can increase the GLP-1 concentration, but there are still many pleiotropic effects of DPP-4 inhibitors independent of GLP-1. These directly increase natriuresis by downregulating the expression of NHE3 [24] and increasing the secretion of brain natriuretic peptide (BNP) or atrial natriuretic peptide (ANP) [24,25]. Additionally, DPP-4 inhibitors exhibit a direct anti-inflammatory effect by inhibiting the DPP-4-mediated degradation of some peptides, including BNP, ANP, stromal cell-derived factor (SDF)-1α, neuropeptide Y, and meprin β [24,26]. In an animal study, sitagliptin was found to suppress the secretion of inflammatory cytokines and apoptosis, thereby preventing glomerular and tubular atrophies [27]. Another study conducted in a streptozotocin-induced diabetic rat model reported that linagliptin was effective in inhibiting kidney fibrosis and restoring renal function [28]. Taken together, these results suggest that DPP-4 inhibitors may have a renoprotective effect. However, the animal studies may not necessarily translate to humans. More clinical studies are needed to clarify the mechanism of the effects of DPP-4 inhibitors on renal function.

The results of clinical studies assessing the effects of DPP-4 inhibitors on renal outcomes were inconsistent. TECOS (Trial Evaluating Cardiovascular Outcomes with Sitagliptin) reported that the median UACR value was marginally lower in the sitagliptin-treated group with a small decline in eGFR than in the placebo group [29]. SAVOR-TIMI 53 (Saxagliptin Assessment of Vascular Outcomes Recorded in Patients with Diabetes Mellitus—Thrombolysis in Myocardial Infarction 53) revealed that the improvement of deterioration of UACR occurred more frequently in the saxagliptin-treated group, regardless of the baseline status of albuminuria (*p* = 0.021, *p* < 0.001, and *p* = 0.049 for individuals with baseline UACR < 30 mg/g, UACR ≥ 30 mg/g to < 300 mg/g, and UACR ≥ 300 mg/g, respectively). Furthermore, the reduction in UACR caused by saxagliptin was not correlated to the reduction of HbA1c, which may be explained by its activity independent of glycemic control. Nevertheless, the changes in eGFR were similar in the saxagliptin and placebo groups [30]. Another study, CARMELINA (Cardiovascular and Renal Microvascular Outcome Study With Linagliptin), showed that linagliptin slowed the progression of albuminuria compared with the placebo (HR 0.86, 95%CI: 0.78–0.95, *p* = 0.003), without significantly different risks of the secondary kidney composite outcome, comprising sustained ESRD, death due to renal failure, or sustained decrease of ≥ 40% in eGFR from baseline, between the two groups [31].

Two systemic review and meta-analysis studies found that DPP-4 inhibitors significantly reduced the risks of developing albuminuria and worsening albuminuria. Moreover, although DPP-4 inhibitors were associated with a greater reduction in eGFR compared with placebo or other anti-hyperglycemic drugs, the risk of ESRD was not different between two groups [32,33]. Our findings contradict previous results [5,10,30,32,33]. Another retrospective study did show results that were consistent with our findings, reporting a significantly lower risk of eGFR decline by ≥ 30% in the DPP-4 inhibitor treatment group [34]. These results may be attributed to the shorter follow-up durations in the above studies, ending before significant eGFR deterioration had occurred. Additionally, we used real-world data in our study, which may be closer to real patients than randomized controlled trials (RCTs). We also excluded patients using SGLT2 inhibitors, which have strong beneficial effects on renal outcomes, to avoid the interference. The two above meta-analysis studies both involved some RCTs comparing DPP-4 inhibitors and SGLT2 inhibitors. This may have affected the results regarding the effects of DPP-4 inhibitors on renal function.

The risks were significantly lower in patients with normal or mildly impaired baseline renal function, with risk reductions of 69% in patients with eGFR ≥ 90 mL/min/1.73 m^2^ and 59% in those with eGFR ≥ 60 to < 90 mL/min/1.73 m^2^. The findings of the LEADER trial were similar. Individuals with eGFR ≥ 60 mL/min/1.73 m^2^, being treated with liraglutide, had a lower risk of composite renal outcomes [10]. From the above inferences, it can be concluded that when DPP-4 inhibitors are used at an earlier stage of CKD, they are more likely to be able to prevent the progression of eGFR decline.

ACEIs and ARBs have renoprotective effects and can delay CKD progression [35]. However, in this study, patients who received ACEIs or ARBs presented an insignificantly lower risk of eGFR decline. This result may have been because the renoprotective effects of ACEIs or ARBs obscured the benefit of DPP-4 inhibitors on renal function, causing a significant risk reduction in eGFR decline among patients who did not use ACEIs or ARBs instead.

This study had some limitations. First, albuminuria, which is a common presentation of DKD and plays an important role in the development of DKD [1,3], was not evaluated, as the available records had insufficient data. Additionally, the contents of anti-hypertensive agents in both groups and the degree of control of hypertension were not detailed due to the limited records, and these aspects may have some influence on proteinuria as well as on renal function. Second, the investigation period was only 5 years, which may be too short to reflect long-term renal outcomes associated with DPP-4 inhibitors. Third, most patients included in this study had no underlying diseases, except for hyperlipidemia and hypertension, with mildly impaired renal function and HbA1c near the glycemic target of 7% [36]; therefore, the results may not be applicable to less healthy patients with DM, who account for a large proportion of patients worldwide. Lastly, this is a retrospective study performed at a medical center, which limits the generalization of its findings. Therefore, further studies are needed.

## 5. Conclusions

In conclusion, the use of DPP-4 inhibitors, alone or in combination with other glucose-lowering agents for glycemic control, was correlated with lower risk of eGFR decline in patients with type 2 DM. 

## Figures and Tables

**Table 1:** Demographic and baseline clinical characteristics of the patients.

	DPP-4 Inhibitor (*n* = 1101)	Non-DPP-4 Inhibitor (*n* = 1101)	*p*-Value
Age (years) ^a^	63.18 ± 11.18	63.43 ± 12.62	0.623
Gender [*n* (%)]			
Female	505 (45.87)	507 (46.05)	0.932
Male	596 (54.13)	594 (53.95)	
Baseline eGFR (mL/min/1.73 m^2^) ^a^	77.84 ± 27.56	77.60 ± 27.19	0.838
Baseline HbA1c (%) ^a^	7.59 ± 1.24	7.63 ± 1.47	0.445
Underlying comorbidities [*n* (%)]			
Coronary artery disease	175 (15.89)	165 (14.99)	0.555
Heart failure	67 (6.09)	63 (5.72)	0.718
Cerebrovascular disease	230 (20.89)	240 (21.80)	0.603
Hyperlipidemia	640 (58.13)	636 (57.77)	0.863
Hypertension	809 (73.48)	825 (74.93)	0.436
Gout	159 (14.44)	146 (13.26)	0.423
Obesity	58 (5.27)	61 (5.54)	0.777
Prior medications [*n* (%)]			
ACEIs or ARBs	570 (51.77)	541 (49.14)	0.216
Direct renin inhibitors	13 (1.18)	11 (1.00)	0.681
Statins	532 (48.32)	507 (46.05)	0.286
Antihypertensive agents	748 (67.94)	756 (68.66)	0.714
Anti-gout drugs	114 (10.35)	115 (10.45)	0.944

^a^ Data = mean ± SD; DPP-4 inhibitor: dipeptidyl-peptidase 4 inhibitor; eGFR: estimated glomerular filtration rate; HbA1c: glycohemoglobin; ACEIs: angiotensin converting enzyme inhibitors; ARBs: angiotensin receptor blockers.

**Table 2:** Renal outcomes in DPP-4 inhibitor group and non-DPP-4 inhibitor group.

	DPP-4 Inhibitor (*n* = 1101)	Non-DPP-4 Inhibitor (*n* = 1101)	*p*-Value
eGFR decline of ≥ 30% [*n* (%)] ^a^	111 (10.08)	178 (16.17)	<0.001
Time to eGFR decline of ≥ 30% (year) ^a,b^	2.84 ± 1.60	1.96 ± 1.30	<0.001

^a^ Decline from baseline; ^b^ data = mean ± SD; DPP-4 inhibitor: dipeptidyl-peptidase 4 inhibitor; eGFR: estimated glomerular filtration rate.

## Data Availability

The data presented in this study are available on request from the corresponding author. The data are not publicly available due to privacy.
